# Immunotherapy in Soft Tissue Sarcomas—An Ongoing Quest

**DOI:** 10.3390/cells15141297

**Published:** 2026-07-21

**Authors:** Silvana Cobeña, Miguel Esperança-Martins, António Syder Queiroz, Cecília Melo-Alvim, Luís Costa

**Affiliations:** 1 Clínica Universitária de Oncologia Médica, Faculdade de Medicina da Universidade de Lisboa, 1649-028 Lisboa, Portugal; silvanacobena@edu.ulisboa.pt (S.C.); cecilia.moreira@ulssm.min-saude.pt (C.M.-A.); luis.costa@ulssm.min-saude.pt (L.C.); 2 Medical Oncology Department, Unidade Local de Saúde de Santa Maria, 1649-035 Lisboa, Portugal; antonio.queiroz@ulssm.min-saude.pt; 3 Oncology Translational Laboratory, Gulbenkian Institute for Molecular Medicine CARE, 1649-028 Lisboa, Portugal

**Keywords:** soft tissue sarcomas, immunotherapy, immune checkpoint inhibitor, adoptive cell therapy, tumor microenvironment, immune responsiveness

## Abstract

**Highlights:**

**What are the main findings?**

- Immunotherapy efficacy in soft tissue sarcomas is limited by low tumor mutational burden, rare microsatellite instability, heterogeneous antigen expression, impaired antigen presentation, and highly diverse immune microenvironments.
- Tumor microenvironment composition, particularly B-cell-rich tertiary lymphoid structures, effector T-cell infiltration, antigen presentation capacity, and myeloid immune contexture, emerges as a major determinant of immunotherapy responsiveness in soft tissue sarcomas.

**What are the implications of the main findings?**

- Biomarker-guided patient selection should move beyond isolated markers such as PD-L1 or tumor mutational burden and integrate intrinsic subtype-specific features, molecular signatures, immune ecosystem profiling, and spatial microenvironmental features.
- Rational strategies that enhance tumor immunogenicity; remodel immune-suppressive microenvironments; combine checkpoint blockade with radiotherapy, chemotherapy, anti-angiogenic agents, epigenetic modulation, or oncolytic viruses; or employ adoptive cell therapies may expand the benefit of immunotherapy in selected soft tissue sarcoma subtypes.

**Abstract:**

Soft tissue sarcomas (STSs) are rare and heterogeneous mesenchymal malignancies characterized by diverse molecular profiles and immune landscapes. Although immunotherapy has revolutionized the treatment of specific solid tumors, its efficacy in STSs remains limited and variable across histotypes. This review explores the panorama of biomarkers of immunotherapy sensitiveness in STSs, with particular emphasis on tumor-intrinsic features and on tumor microenvironment (TME) signatures. Current evidence highlights low tumor mutational burden, rare microsatellite instability, heterogeneous antigen expression, and epigenetic suppression of antigen presentation as hallmarks of the immune resistance that is characteristic of many STSs. However, growing evidence underlines TME composition as a major determinant of response to different types of immunotherapy. Indeed, the presence of B-cell-rich tertiary lymphoid structures and certain traits of adaptive immune responses are provenly associated with enhanced sensitivity to immunotherapy and enhanced outcomes. We further discuss emerging strategies aimed at enhancing STS immunogenicity, either by increasing intrinsic tumor immunogenicity or remodeling TME composition and functional profile. Collectively, the available data support a paradigm shift from a sarcoma cell-centered approach toward a multi-compartment TME-including strategy, providing a framework for the development of more effective and personalized immunotherapeutic strategies in STS.

## 1. Introduction

Sarcomas comprise a highly heterogeneous and fragmented group of rare malignancies, encompassing over 100 histopathological subtypes with diverse molecular drivers, clinical behaviors, and therapeutic sensitivities [1,2,3]. This marked heterogeneity, combined with their low incidence (approximately 1–2% of adult cancers), represents a major barrier to drug development, as individual subtypes rarely achieve sufficient representation in preclinical studies and clinical trials [4,5]. Current classification systems remain largely based on cytoarchitectural features, morphological characteristics and presumed lines of differentiation, an approach that is inherently indirect, imprecise and associated with significant major diagnostic discordances even in expert centers [3,6]. Consequently, therapeutic strategies have historically followed a largely “one-size-fits-all” paradigm, particularly in advanced disease, despite the existence of selected histotype-specific approaches. Management of localized soft tissue sarcomas (STSs) relies on multimodal treatment combining surgery and radiotherapy, with chemotherapy reserved for high-risk cases [7,8]. In contrast, systemic therapy remains the cornerstone for advanced disease, with anthracycline-based regimens as first-line treatment and a limited number of subtype-specific targeted agents available [9]. Despite these strategies, outcomes remain suboptimal, with recurrence rates of 20–40% in localized disease and median overall survival of 12–20 months in metastatic settings [10,11].

In this context, the incorporation of immunotherapy into the sarcoma treatment armamentarium has been limited, with only a small number of immune-based therapies being approved for selected indications. These include immune checkpoint inhibitors (ICIs) such as atezolizumab for alveolar soft part sarcoma (ASPS) [12]. The modest efficacy observed across most sarcoma subtypes reflects multiple intrinsic barriers. Biologically, many sarcomas exhibit low tumor mutational burden (TMB), infrequent microsatellite instability (MSI), variable levels of expression of immune checkpoint receptors (such as PD-L1), and limited expression of immune-targetable neoantigens, contributing to reduced immunogenicity [11,13]. Additionally, sarcomas’ TME is frequently immunosuppressive, characterized by low effector T-cell infiltration, presence of regulatory immune populations, and stromal features that hinder immune activation [9,13]. Finally, conceptually, sarcomas’ extreme heterogeneity further complicates patient stratification and trial design, while their rarity limits the generation of robust clinical evidence. Together, these factors contribute to sarcomas’ resistance to immunotherapy, restricting its broader clinical applicability in sarcomas.

Given these challenges, there is a critical need to better define the determinants of immune responsiveness and to develop innovative strategies capable of overcoming these biological and clinical barriers. In this review, we aim to synthesize current evidence on immunotherapy in sarcomas, highlighting emerging approaches developed with the objective to enhance sarcomas’ immunogenicity, allowing their combination with different immune-based therapies across distinct sarcoma subtypes.

## 2. Review Methodology and Scope

This article was designed as a narrative literature review, aiming to provide a comprehensive and up-to-date overview of the current landscape of immunotherapy in STSs, with particular emphasis on tumor-intrinsic determinants and tumor microenvironmental signatures of immune sensitivity/resistance, subtype-specific patterns of response, and emerging strategies to enhance immune responsiveness.

A focused literature search was conducted between July 2025 and May 2026 using major biomedical databases, including PubMed/MEDLINE, Scopus, Web of Science, and Google Scholar. Search terms were adapted according to each topic and included combinations of “soft tissue sarcoma”, “sarcoma”, “tumor microenvironment”, “tumor-associated macrophages”, “tertiary lymphoid structures”, “predictive biomarkers”, “cell states”, “immune classes”, immunotherapy”, “immune checkpoint inhibitor”, “adoptive cell therapy”, “CAR-T”, “TCR-T”, “radiotherapy”, and “combination therapy”. The review was structured according to predefined thematic domains that reflected the conceptual framework of this work, including the conceptual biological basis of the employment of immunotherapy in sarcomas, TME characterization, mechanisms of immune responsiveness and resistance, predictive biomarkers, therapeutic strategies, and future perspectives. For each topic, priority was given to high-quality evidence, including randomized clinical trials, prospective studies, landmark translational studies, meta-analyses, systematic reviews, and high-impact narrative reviews. Recently published studies were preferentially included whenever they provided relevant advances or updated clinical evidence. Articles were included if they addressed immunotherapy, immune biology, tumor microenvironment, biomarkers, or therapeutic strategies in STS. Studies exclusively focused on bone sarcomas, non-English publications, conference abstracts lacking sufficient methodological detail, and articles with limited relevance to the objectives of this review were excluded.

Given the narrative nature of this review, article selection was based on scientific relevance, methodological quality, and contribution to the current understanding of the field rather than on predefined systematic review criteria.

## 3. Conceptual Challenges

### 3.1. Low Tumor Mutational Burden and Low Incidence of Microsatellite Instability

**Figure 1:**
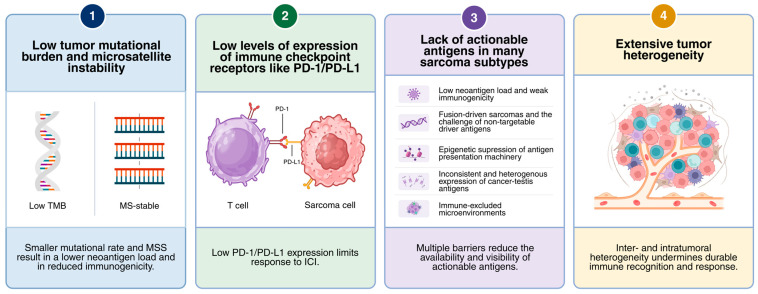
Conceptual challenges limiting the efficacy of immunotherapy in sarcomas. ICI—immune checkpoint inhibitor; PD-1—programmed cell death protein 1; PD-L1—programmed death-ligand 1; TMB—tumor mutational burden.

STSs are mesenchymal malignancies typically characterized by a low TMB, with median values around 1–2 mutations per megabase, and a very low incidence of MSI, observed in less than 1% of cases [14,15,16] (Figure 1). TMB and MSI are established predictive biomarkers of response to ICIs, and their scarcity in STSs contributes to the limited immunogenicity of these tumors. Hypermutated sarcomas (TMB ≥ 10–20 mut/Mb) are exceedingly rare (<1%), and MSI-high phenotypes are similarly uncommon, being occasionally reported in specific histotypes such as uterine leiomyosarcoma (LMS), angiosarcoma, undifferentiated pleomorphic sarcoma (UPS), malignant peripheral nerve sheath tumor (MPNST), or in patients with hereditary cancer syndromes like Lynch syndrome [15,17]. Consistently, mismatch repair (MMR) deficiency is also extraordinarily rare (~1%), largely restricted to sarcoma patients with a genetic predisposition or to patients with sarcomas with myogenic differentiation [17]. Overall, the limited mutational landscape of STS results in a reduced neoantigen repertoire, contributing to an “immune-cold” phenotype and contributes to the modest clinical efficacy of immunotherapy compared with highly immunogenic tumors such as melanoma or non-small cell lung cancer [16,18].

### 3.2. Low Levels of Expression of Immune Checkpoint Receptors (Such as PD-1/PD-L1)

Expression of conventional immune checkpoints receptors such as PD-1/PD-L1 in STS is generally low and highly heterogeneous across histotypes, limiting their utility as standalone prognostic or predictive biomarkers. Large immunohistochemical studies show that PD-1 and PD-L1 positivity occurs in only a minority of cases and does not consistently correlate with survival outcomes. Notably, alternative immune checkpoints, including LAG-3 and TIM-3, are more frequently expressed and often co-expressed with PD-1, particularly in non-translocation-associated sarcomas, suggesting that immune suppression in STS is typically multiaxial rather than predominantly PD-1/PD-L1-driven [19,20,21,22,23].

Given the rarity, heterogeneity, and dynamic regulation of PD-1/PD-L1 expression, increasing evidence supports its interpretation within a broader immunogenomic context. Genomically complex sarcomas, such as UPS, LMS, dedifferentiated liposarcoma (DDLPS), myxofibrosarcoma (MFS), and angiosarcoma, are characterized by chromosomal instability and alterations in p53 and Rb pathways and may occasionally harbor actionable features such as increased TMB or CD274 (PD-L1) amplification, which can influence immunogenicity and therapeutic vulnerability [24]. PD-L1 expression varies throughout different histotypes and is closely linked to TME profile, often reflecting an inducible, inflammation-driven state rather than a stable tumor-intrinsic feature. Accordingly, higher PD-L1 expression is more frequently observed in immune-infiltrated, genomically complex subtypes, where it correlates with tumor-infiltrating lymphocyte (TIL) density and IFN-γ–associated inflammatory programs, while translocation-driven “immune-cold” sarcomas are typically PD-L1-negative. Importantly, PD-L1 expression may increase in metastatic or recurrent lesions, highlighting temporal and spatial variability, which makes single-biopsy interpretation at least limited [19,21,23,25,26].

Clinically, responses to PD-1/PD-L1 blockade are modest and non-uniform, reflecting underlying biological heterogeneity. Greater sensitivity is observed in more inflamed subtypes, particularly UPS, and in specific entities such as ASPS, the single sarcoma histotype for which a PD-1/PD-L1 axis modulator, Atezolizumab, is approved in monotherapy [23,24,25,27,28]. Overall, PD-L1 expression alone has limited predictive value but gains relevance when integrated with additional parameters such as TILs, T-cell receptor (TCR) clonality, TLSs, TMB, and genomic context [28]. High clonality and checkpoint expression define inflamed subtypes more likely to respond to immunotherapy, whereas immune-cold, translocation-driven sarcomas are typically resistant. The dynamic regulation of PD-L1, including gene amplification and IFN-γ-mediated upregulation, further underscores its role as a context-dependent marker of tumor–immune interaction rather than a static biomarker [22,26,27].

### 3.3. Lack of Actionable Antigens in Many Soft Tissue Sarcoma Subtypes

The absence of consistently actionable tumor antigens across most histopathologic subtypes represents a major conceptual difficulty in developing effective immunotherapies for STS. While highly mutagenic epithelial cancers generate abundant neoantigens capable of eliciting strong T-cell responses, STSs are generally characterized by low TMB, minimal neoantigen production, and frequent immune-cold microenvironments, limiting the pool of targetable antigens for checkpoint inhibitors, vaccines, engineered TCRs, and CAR-T cell therapies. This challenge is multifactorial and should be examined across several dimensions: intrinsically low neoantigen load and weak immunogenicity; fusion-driven sarcomas in which oncogenic drivers are poorly targetable at the peptide level and by currently known ICI-based therapies; epigenetic suppression of antigen presentation machinery; inconsistent and heterogeneous expression of CTAs across the whole pool of sarcoma histotypes; immune-excluded microenvironments that limit effective antigen recognition; and rare biologic exceptions that illustrate contexts in which antigen-directed immunotherapy can succeed [29,30].

#### 3.3.1. Low Neoantigen Load and Weak Immunogenicity

Across multiple genomic studies, it has been demonstrated that most STSs carry few nonsynonymous mutations, DNA alterations that result in the modification of the amino acid sequence of the encoded protein, which give rise to neoantigens since they alter protein structure [31]. Unlike cancers such as melanoma or lung cancer—where a high TMB produces many neoantigens, which are strongly linked to good responses to immunotherapy—sarcomas do not seem to provide enough recognizable targets. Thus, the antigen landscape in STS is scarce, limiting the ability of cytotoxic T cells to detect and attack tumor cells and reducing the potential effectiveness of ICI-based therapies overall [31,32]. Even in the small subset of “immune-hot” sarcomas, the level of antigenicity varies between patients and rarely results in consistent or long-lasting clinical responses [33].

#### 3.3.2. Fusion-Driven Sarcomas and the Challenge of Non-Targetable Driver Antigens

Approximately 20–30% of STSs are driven by pathognomonic chromosomal translocations, generating fusion oncoproteins such as SS18–SSX (synovial sarcoma) and PAX3/7–FOXO1 (alveolar rhabdomyosarcoma). Although these fusions are highly specific to the tumor, they result in the production of fusion proteins that remain in the intracellular compartment, not being displayed on the cell surface, and that are therefore poorly accessible to standard antigen-directed therapies [34]. Their intracellular localization limits the feasibility of the development of CAR-T directed to these fusion proteins, and despite their immunogenic potential as peptide antigens, epitope presentation is often inefficient or rapidly suppressed within the TME [33,34].

#### 3.3.3. Epigenetic Suppression of Antigen Presentation

Epigenetic dysregulation represents a major mechanism of immune evasion in STS, impairing antigen presentation through DNA hypermethylation, histone modifications (notably H3K27me3), chromatin remodeling alterations, and silencing of key regulators such as NLRC5 and CIITA [35,36]. These alterations frequently result in downregulation or loss of HLA class I expression across multiple sarcoma subtypes, including myxoid liposarcoma, UPS, and fibrosarcoma, thereby limiting effective antigen presentation. Loss of HLA polymorphic determinants further enables tumor escape from both T-cell and natural killer cell surveillance, and profound HLA-I deficiency has been shown to impair cytotoxic responses even in the context of immune checkpoint blockade [37,38].

At a mechanistic level, distinct epigenetic programs converge to suppress antigen presentation in a subtype-specific manner. In MPNST, loss of PRC2 function (via EED or SUZ12 mutations) leads to compensatory epigenetic reprogramming, including DNA methylation and H3K36me2 deposition, ultimately reducing interferon signaling and MHC expression, effects that may be reversible through NSD2 inhibition [39,40]. In contrast, translocation-driven sarcomas such as synovial sarcoma (SS) exhibit fusion protein-mediated disruption of SWI/SNF chromatin remodeling complexes, resulting in repression of differentiation programs alongside derepression of cancer–testis antigens (CTAs), thereby generating a distinct antigenic landscape rather than complete loss of antigen presentation [41,42,43]. Additionally, direct epigenetic silencing of antigen presentation regulators, such as NLRC5 and CIITA, further contributes to immune escape, as demonstrated in rhabdomyosarcoma and other pediatric sarcomas, where combined epigenetic therapies can restore MHC expression [44,45].

#### 3.3.4. Inconsistent Expression of CTAs

CTAs have emerged as promising targets for engineered TCR therapies. However, their expression in STS is highly heterogeneous, often limited, and inconsistently associated with clinical outcomes [31,32]. The prevalence of CTAs such as PRAME, NY-ESO-1, MAGE-A4, and SSX2 varies according to sarcoma molecular subtype and tumor-specific characteristics, with higher expression typically observed in translocation-associated tumors [46]. SS and myxoid/round cell liposarcoma (MRCL) represent the most CTA-enriched subtypes, with frequent expression of NY-ESO-1 and MAGE-A4, while PRAME is detected in approximately 10–48% of STSs overall and SSX2 is particularly prevalent in SS [46,47]. Nevertheless, even within these enriched subtypes, antigen expression is not universal. For example, in metastatic SS, although PRAME expression reaches up to 91%, a significant proportion of tumors lack NY-ESO-1 and MAGE-A4, and some are negative for all evaluated CTAs, underscoring incomplete antigen coverage [48].

Co-expression patterns further highlight this variability, as some tumors express multiple CTAs while others express only one or none. Although co-expression may expand therapeutic options, the clinical efficacy of TCR-based therapies primarily depends on strong and homogeneous expression of a single target antigen rather than antigen multiplicity. Consequently, CTA-directed immunotherapy is restricted to a molecularly defined subset of patients and requires precise antigen profiling prior to treatment [46,49]. Overall, despite their therapeutic potential in selected subtypes, the heterogeneous and often incomplete expression of CTAs across and within STSs remains a major limitation to their broad clinical applicability [33,46,49].

#### 3.3.5. Immune-Excluded Microenvironments Limit Antigen Utility

In addition to antigen scarcity, many STS subtypes are characterized by low CD8^+^ T-cell infiltration and abundant immunosuppressive myeloid populations, particularly M2-polarized tumor-associated macrophages (TAMs), along with regulatory T cells (Tregs), cancer-associated fibroblasts (CAFs), mesenchymal stromal cells (MSCs) and myeloid-derived suppressor cells (MDSCs). Together, these components establish an immune-suppressed TME, which is going to be characterized in greater detail in subsequent sections. Thus, even when tumor antigens are theoretically present, their therapeutic relevance is undermined by conditions that prevent effective antigen recognition or T-cell infiltration. Such mechanisms include downregulation of MHC class I molecules, loss of heterozygosity at HLA loci, impaired antigen processing machinery, and dysfunctional interferon signaling pathways, all of which limit productive cytotoxic T-cell engagement [29,30].

### 3.4. Extensive Tumor Heterogeneity

The rarity and extensive biological, genomic, and immunological heterogeneity of sarcomas underlie their highly variable and often limited response to immunotherapy [31,32]. STSs can be broadly classified into two genomic groups, as previously mentioned: translocation-associated sarcomas with simple karyotypes (e.g., SS and MRCL) and complex genomic sarcomas characterized by chromosomal instability and copy-number alterations (e.g., UPS, DDLPS, and LMS) [33,50]. These groups differ substantially in TMB, gene-fusion architecture, antigenic diversity, and immune visibility [32]. Translocation-driven sarcomas typically harbor a single dominant oncogenic event and exhibit a very low TMB, limited neoantigen generation, and minimal immune infiltration, whereas complex genomic sarcomas accumulate widespread alterations that may support more variable immune infiltration and heterogeneous checkpoint and antigen presentation profiles [31,51]. This genomic divergence translates into distinct TMEs, ranging from relatively inflamed phenotypes, with CD8^+^ T-cell infiltration, macrophage involvement, and tertiary lymphoid structures (TLSs) in subtypes such as UPS, MFS, and DDLPS, to predominantly immunosuppressive environments characterized by M2-polarized macrophages, T-cell exclusion, low PD-L1 expression, and defective antigen presentation, which collectively confer resistance to immune checkpoint blockade [30,33,52].

Importantly, substantial heterogeneity also exists within individual histologic subtypes, with molecularly distinct clusters displaying divergent immune signatures, levels of immune infiltration, and checkpoint expression (e.g., PD-1, PD-L1, LAG-3, and IDO1) [31,50,51]. Epigenetic mechanisms, including alterations in chromatin remodeling complexes such as SWI/SNF, DNA methylation patterns, and oncogenic fusions, further diversify immune phenotypes and may generate “immune-hot” niches even within low-TMB tumors [34,53]. In addition, methodological variability in sampling, tissue processing, and biomarker assessment introduces further complexity, limiting cross-study comparability [31]. Altogether, this extensive inter- and intratumoral heterogeneity constrains the development of universal biomarkers and contributes to inconsistent clinical responses, with meaningful benefit from immunotherapy largely restricted to selected subtypes such as ASPS [29,52]. Overcoming these challenges will require histotype-specific strategies supported by integrated genomic and immunologic profiling and deeper mechanistic understanding of sarcoma immune landscapes [34].

## 4. Histotype-Specific Immunotherapy Sensitivity and Responsiveness

A histotype-specific framework is essential because STSs do not behave as single immunologic entities. They encompass distinct immune ecosystems that differ in lymphocyte infiltration, macrophage composition, overall antigenicity, stromal organization, and global responsiveness to different types of immunotherapy [32,54,55].

Broadly, complex genomic sarcomas such as UPS, DDLPS, MFS, and subsets of LMS may contain more immune-infiltrated tumors, although this remains highly variable and often shaped by myeloid and stromal exclusion. In contrast, translocation-driven sarcomas such as SS and MRCL usually have a low TMB and limited endogenous immune priming, but may express cancer–testis antigens that create a therapeutic window for TCR-engineered cellular therapies. ASPS represents a distinct paradigm, showing reproducible sensitivity to PD-1/PD-L1 blockade despite a low TMB, likely reflecting a permissive immune-vascular context rather than conventional neoantigen-driven immunogenicity.

More specifically, UPS may exhibit appreciable T-cell infiltration; significant TLS density; and, theoretically, a higher relative sensitivity to ICI [32,52]. While MFS shares some of the UPS genomic and immunogenic features, it has its own specific immune signatures. Therefore, these two histotypes should not be considered immunologically interchangeable despite their morphological and molecular similarities [56,57]. LPS also displays a relatively inflamed TME. Nevertheless, the responses to PD-1/PD-L1 axis modulation in monotherapy in this specific histotype are variable. Biomarker-guided combinations of PD-1/PD-L1 axis modulators and CDK4/6 or MDM2-targeted therapies may be promising [32]. LMS is itself a particularly heterogenous histotype where immune landscape is concerned. Indeed, there are important molecular and immunological differences between distinct LMS subsets [58]. Angiosarcoma follows the same pattern, also being heterogeneous in terms of the immune sensitivity features concerned across anatomic sites: cutaneous head and neck angiosarcomas frequently exhibit a high TMB, UV mutational signatures, and enrichment in cytotoxic T cells, PD-L1^+^ cells, and elevated tumor inflammation signature scores, providing a strong rationale for checkpoint immunotherapy; by contrast, visceral angiosarcomas tend to be mutationally quiet, with distinct epigenetic and oncogenic profiles (e.g., MYC amplification) that may be more amenable to targeted therapies than to ICI alone [59,60,61]. ASPS, as previously mentioned, represents a notable exception by demonstrating substantially greater sensitivity to PD-1/PD-L1 ICI than the large majority of other STS histotypes [54]. Among translocation-driven sarcomas, SS and MRCL share low immune infiltration and poor responsiveness to ICI, yet their mechanisms of immune evasion are dissimilar. SS generally exhibits low immune infiltration despite expressing highly immunogenic CTAs, making engineered TCR therapies targeting NY-ESO-1 or MAGE-A4 attractive, in opposition to checkpoint blockade alone [29,32]. MRCL evades immune recognition primarily through near-universal downregulation of HLA class I, which impairs antigen presentation to CD8^+^ T cells regardless of CTA expression, and harbors a macrophage-dominated microenvironment with CD163^+^ M2-polarized macrophages associated with a worse prognosis; these features suggest that strategies aimed at restoring MHC-I expression—such as epigenetic modulators—may be required to render MRCL amenable to T-cell-directed therapies [38,62].

Future trials should avoid treating STSs as a homogeneous group and should instead integrate molecular subtype, molecular driver, antigenic targetability, immune infiltration, myeloid/stromal context, and spatial architecture into eligibility and stratification criteria.

Table 1 summarizes histotype-specific immunobiology and immunotherapeutic implications in STS.

## 5. Putting the Spotlight on the Sarcoma Microenvironment: Characterization of Its Cellular Population, Immune Profile and Ecosystem Diversity

### 5.1. Immune Microenvironment in Soft Tissue Sarcomas

**Figure 2:**
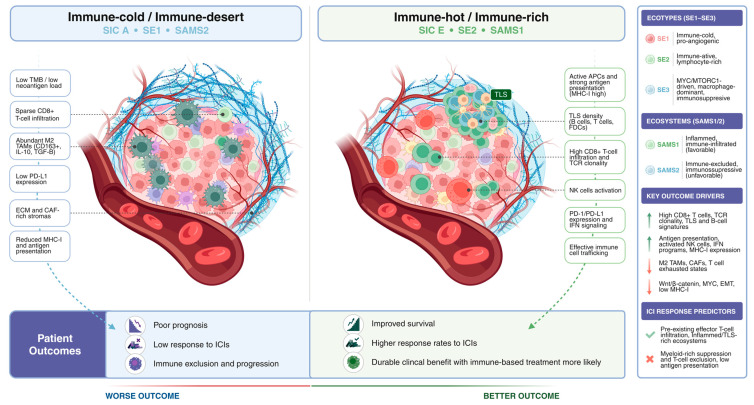
Immune profiles of STS microenvironment. APCs—antigen-presenting cells; CAFs—can-cer-associated fibroblasts; ECM—extracellular matrix; ICIs—immune checkpoint inhibitors; IFN—interferon; NK—natural killer; TAMs—tumor-associated macrophages; TCR—T cell receptor; TLS—tertiary lymphoid structure; TMB—tumor mutational burden.

The immune microenvironment of STSs is highly heterogeneous and represents a major determinant of prognosis and response to immunotherapy [31,32]. Although historically considered “immune-cold,” sarcomas encompass a wide spectrum ranging from immune-desert tumors with minimal lymphocytic infiltration to immune-rich subtypes characterized by organized TLSs and active immune signaling [33,55] (Figure 2). Within this diverse landscape, the myeloid compartment, particularly TAMs, predominates over lymphocytes in most STSs [31]. These TAMs are frequently skewed toward an immunosuppressive M2-like phenotype, marked by CD163 expression and secretion of IL-10 and TGF-β, which suppress cytotoxic T-cell activity, promote angiogenesis, and support tumor progression [50,63]. High densities of M2-polarized macrophages are associated with worse survival and reduced responsiveness to ICIs, while spatial analyses further suggest that perivascular TAMs contribute to immune exclusion by regulating immune cell trafficking [33,51,64].

T-cell infiltration is likewise variable across sarcoma subtypes and is generally modest [25]. Complex genomic sarcomas such as UPS, DDLPS, MFS, and some LMSs tend to exhibit higher CD3^+^ and CD8^+^ T-cell densities and more active antigen presentation profiles, whereas translocation-driven tumors such as SS and Ewing sarcoma are typically characterized by T-cell exclusion and low immunogenicity [31,65]. In particular, UPS may display an “inflamed” phenotype defined by diffuse CD8^+^ infiltration, oligoclonal TCR repertoires, and elevated PD-1/PD-L1 expression, correlated with relatively improved responses to checkpoint blockade. However, even within single histotypes, T-cell density and prognostic significance remain inconsistent, reflecting both biological and methodological heterogeneity [25,63].

B cells and TLSs have emerged as pivotal components of the sarcoma immune microenvironment. While most STSs exhibit limited CD20^+^ B-cell infiltration, a subset of them are associated with TLSs composed of B cells, T cells, and follicular dendritic cells [31,63]. Transcriptomic analyses of the samples included in the SARC028 trial have permitted the identification of five Sarcoma Immune Classes (SIC A–E), with SIC E representing TLS-rich sarcomas associated with the best overall survival and highest response rates to PD-1 blockade [55]. Indeed, TLS-positive sarcomas, more commonly UPS, DDLPS, MFS, and some rhabdomyosarcomas, display superior clinical outcomes, with TLSs outperforming PD-L1 expression and CD8^+^ T-cell density as predictive biomarkers of response to ICI [32,51,55].

Immune checkpoint expression further reflects this heterogeneity. PD-1 and PD-L1 are more commonly upregulated in immune-inflamed, complex genomic sarcomas, whereas many translocation-driven tumors remain PD-L1-negative [50,55]. In addition, alternative checkpoints, including LAG-3, TIM-3, TIGIT, IDO1, and the CD47–SIRPα axis, are frequently co-expressed in immune-high tumors, indicating T-cell exhaustion and macrophage-mediated immune evasion and providing a rationale for combinatorial immunotherapeutic strategies targeting multiple pathways [51,66].

Overall, sarcoma microenvironmental immune composition is both subtype-specific and patient-specific, shaped by the interplay between TAM polarization, T-cell infiltration and exhaustion, B-cell/TLS organization, and checkpoint co-expression [51,55,63]. This complexity underlies the limited and heterogeneous responses to immunotherapy, emphasizing the need for integrated biomarker-driven stratification and rational combination strategies aimed at enhancing antigenicity while overcoming myeloid-driven immunosuppression [32,51,65].

### 5.2. Cell States, Ecotypes and Ecosystems of Sarcomas: Definition and Validation

STS microenvironments are organized into discrete cell states and higher-order ecotypes that portray the functional architecture of the TME [67]. Across malignant, immune, and stromal compartments, 23 sarcoma-specific cell states have been identified, characterized, and explored with the purpose of understanding if these states represent truly stable transcriptional programs. These sarcoma-specific cell states range from proliferative or quiescent malignant cell populations to activated or exhausted immune cell types, such as specific macrophage phenotypes. Three sarcoma ecotypes (SE1-SE3) have been identified: 1. SE1: an immune-cold, pro-angiogenic ecotype; 2. SE2: an immune-active, lymphocyte-rich ecotype; 3. SE3: a MYC/MTORC1-driven, macrophage-dominated immunosuppressive ecotype.

These ecotypes have a strong prognostic and predictive value, which will be explored in the next section [67].

Furthermore, using integrated transcriptomic, epigenomic, and different types of single-cell analyses, two robust molecular ecosystems have been labelled: SAMS1 and SAMS2 [68]. On the one hand, SAMS1 represents an inflamed, immune-infiltrated ecosystem, characterized by abundant T and NK cells, active interferon signaling, and enhanced antigen presentation. On the other hand, SAMS2 represents an immune-excluded, immunosuppressive ecosystem, enriched for M2-like macrophages, CAFs, and malignant states driven by Wnt- β-catenin, EMT, and metabolic reprogramming, with reduced MHC expression and diminished cell–cell communication [68].

### 5.3. Association Between Cell-State Abundance, Ecotypes and Ecosystem Profiles, and Patient Outcomes in Sarcomas

The cellular composition of tumor and immune cell states within the sarcoma microenvironment plays a central role in determining prognosis and therapeutic responsiveness [69]. Different analyses have demonstrated that distinct malignant, immune, and stromal ecotypes carry prognostic significance and predictive value (in terms of immune-based therapies’ efficacy). For instance, immune-active ecotypes such as SE2, enriched in cytotoxic CD8^+^ T cells, B cells, and antigen-presenting cells, are associated with significantly improved overall survival in localized disease, whereas SE3 correlates with poorer baseline prognosis but paradoxically increased responsiveness to ICIs, highlighting the complex interplay between immune suppression and therapeutic vulnerability [67].

Beyond cellular composition, the functional state of immune populations critically shapes outcomes. High densities of CD8^+^ T cells, increased TCR clonality, and strong antigen presentation signatures are associated with improved survival, particularly in subtypes such as UPS [69]. Similarly, enrichment in activated NK-cell states, MHC-I-high profiles, and interferon-responsive programs correlates with favorable outcomes, whereas exhausted T-cell states and myeloid-driven immunosuppressive niches predict early progression [70]. Importantly, TMB modulates these associations, as cytolytic immune signatures (e.g., CD8^+^ T cells and Th1) confer survival benefit primarily in TMB-high tumors. On the other hand, a B-cell/plasma-cell-associated signature remains prognostically favorable even in TMB-low settings, underscoring the relevance of humoral immunity [18].

At a broader ecosystem level, sarcomas enriched in immune-active cell states, such as those classified within the SAMS1 ecosystem, characterized by high densities of CD8^+^ T cells, dendritic cells, and NK cells, demonstrate significantly improved survival compared with SAMS2 tumors, which are dominated by immunosuppressive myeloid populations, CAFs, and EMT-like malignant states [68]. These unfavorable ecosystems are associated with activation of pathways such as Wnt/β-catenin and MYC, which contribute to immune exclusion and poor prognosis. Consistently, transcriptomic risk models based on immune-related gene expression further confirm that immune-infiltrated and antigen-presenting cell states are robust predictors of improved survival across sarcoma cohorts [71].

Complementarily, the Sarcoma Immune Classes (SIC A–E) provide an additional framework to stratify tumors according to their immune microenvironment composition. SIC E tumors, enriched in B cells, TLSs, and coordinated adaptive immune responses, are associated with the most favorable survival outcomes and the highest responsiveness to immune checkpoint blockade, whereas immune-desert classes such as SIC A exhibit poor immune infiltration and worse prognosis [18,55,68]. Together, these findings reinforce the concept that the sarcoma TME is a critical determinant of clinical outcome and highlight the importance of integrating immune ecosystem profiling into prognostic stratification and therapeutic decision-making.

### 5.4. Predicting Response to Immune Checkpoint Inhibitors Based on Microenvironmental Characteristics

Response to ICIs in STS remains limited and is largely determined by the pre-existing TME composition and functional landscape rather than by histotype or PD-L1 expression alone [32,65,72]. Accumulating evidence supports the concept that clinical benefit occurs mainly in tumors that are already immunologically “primed” with active yet partially suppressed adaptive immune response signatures. In this context, baseline effector T-cell infiltration and activation state represent the most consistent predictors of response to PD-1/PD-L1 blockade. In the SARC028 trial, responders to pembrolizumab exhibited higher densities of intratumoral CD3+ and CD8+ T cells, including antigen-experienced PD-1+ effector-memory populations, which were also associated with improved recurrence-free survival [72]. Prototypical “inflamed” subtypes such as UPS display high antigen presentation gene expression, PD-1/PD-L1 upregulation, and oligoclonal TCR repertoires, whereas immune-excluded tumors show limited responsiveness even when treated with combinations such as radiotherapy plus immunotherapy, despite partial immune remodeling [12,25].

B-cell-enriched TLSs have emerged as some of the most robust predictors of ICI sensitiveness in sarcomas. SIC-E tumors, characterized by the coexistence of B-cell and adaptive immune activity signatures, have shown response rates of approximately 50% to pembrolizumab in the SARC028 trial, significantly outperforming other immune classes in terms of overall response rate [55]. Subsequent analyses have consistently associated the presence and density of TLSs with improved progression-free survival (PFS) and higher overall response rates, surpassing CD8^+^ T-cell density alone as a predictive biomarker, although some variability across cohorts persists [32,51,53,73]. In parallel, the myeloid compartment plays a critical modulatory role: enrichment of PD-L1^+^ TAMs has been linked to enhanced response rates, suggesting active immune engagement even in PD-L1-negative tumor cells, whereas dominance of M2-polarized TAMs, elevated neutrophil-to-lymphocyte ratios, and poor Lung Immune Prognostic Index (LIPI) scores are associated with inferior outcomes [53,72,73]. Spatial organization further influences response, as exemplified by UPS, where T cells and macrophages are interdigitated, in contrast to rhabdomyosarcoma, where T cells remain spatially excluded despite similar abundance [64].

Overall, ICI responsiveness in STS is driven by the convergence of effector T-cell infiltration, B-cell/TLS niches, functional antigen presentation, and a non-immunosuppressive myeloid environment, rather than by isolated biomarkers such as PD-L1 expression alone [32,53].

## 6. Biomarker Maturity and Clinical Validation in Sarcoma Immunotherapy

A practical interpretation of immunotherapy biomarkers in STS requires a distinction between clinically established or actionable biomarkers, promising translational biomarkers, and exploratory biomarkers that remain hypothesis-generating. At present, no single biomarker reliably predicts response across all STS histotypes. TMB-high, MSI-high, and MMR-deficient phenotypes have tumor-agnostic therapeutic relevance but are rare in STS and therefore explain only a small fraction of immunotherapy-responsive cases. PD-L1 expression has limited standalone predictive value because it is spatially heterogeneous, dynamically inducible, histotype-dependent, and inconsistently associated with response. In contrast, B-cell-rich tertiary lymphoid structures and immune-inflamed transcriptomic classes have emerged as being among the most promising predictors of benefit from PD-1 blockade in sarcoma, but their clinical implementation is constrained by a lack of standardized assessment, variable spatial distribution, limited prospective validation, and uncertain performance across histotypes. More recent frameworks, including sarcoma ecotypes, SAMS ecosystems, metabolic signatures, and spatial immune profiling, provide mechanistic insight into immune permissiveness or exclusion, but will remain exploratory until prospectively tested and harmonized across platforms.

Table 2 displays stratification of STS immunotherapy biomarkers by level of clinical validation.

The principal limitation is the absence of standardized assays, reproducibility across cohorts, and prospective clinical qualification.

While PD-L1 immunohistochemistry remains the most widely used biomarker in clinical practice and TMB has regulatory approval in selected settings, both exhibit important technical and biological limitations. In sarcomas specifically, PD-L1 expression is dynamic, susceptible to inducible regulation and prone to spatial/temporal variability across primary and metastatic lesions, features that further limit the reliability of single-timepoint, single-biopsy assessment of PD-L1 expression levels. Moreover, PD-L1 expression is typically very low (median 0%) and TMB ≥10 mut/Mb is found in only ~3% of patients; although PD-L1 positivity (≥1%) enriches for response, the extremely low prevalence of both biomarkers limits their utility as universal selection tools, and their low mutual correlation suggests that they capture distinct dimensions of tumor immunogenicity [74,75].

Among translational biomarkers, TLSs have consistently emerged as one of the strongest predictors of response to immune checkpoint blockade, reflecting the presence of organized local antitumor immune structures. The PEMBROSARC trial provided the first prospective evidence that TLS-based patient selection substantially improves ICI outcomes in STS, with a 6-month non-progression rate of 40% and an ORR of 30% in TLS-positive patients versus 4.9% and 2.4% in unselected cohorts [28]. Exploratory analyses identified ntratumorall plasma cell and B-cell abundance as being significantly associated with improved outcome, consistent with the finding that B cells represent the strongest prognostic factor in STS independently of CD8^+^ T-cell content, while TLS maturation status—specifically the presence of germinal center B cells versus immunosuppressive M2-like macrophages in immature TLSs—has emerged as an additional layer that may refine predictive value beyond simple TLS presence or absence [54,76]. Despite their promise, TLSs and related immune transcriptomic classifications carry important limitations that currently preclude their routine clinical use. First, there is no consensus, standardized methodology for TLS identification and quantification in STS—approaches vary from qualitative histologic assessment to gene-expression signatures, limiting cross-study comparability and reproducibility. Second, TLS distribution within tumors is spatially heterogeneous, and small biopsy specimens may under- or over-represent true TLS density, favoring whole-section or multi-region sampling that is not always feasible in clinical practice. Third, the prognostic and predictive significance of TLSs appears to vary across histotypes, being most consistently reported in complex genomic, T-cell-infiltrated subtypes (UPS, DDLPS, and MFS) and less well characterized in translocation-driven, immune-excluded sarcomas, which limits the generalizability of a single TLS-based algorithm across the full spectrum of STSs [77]. Likewise, immune transcriptomic classifications, including T-cell-inflamed gene-expression signatures and immune classes, frequently outperform individual biomarkers such as PD-L1 in retrospective analyses. However, their reproducibility across independent datasets, histological subtypes, and prospective clinical trials remains insufficient for routine implementation [78,79,80]. A notable exception is the SIGNIOS signature (CD86, CHI3L1, CXCL10, CXCL9, LAG3, NR4A1, and VCAM1), recently validated in the independent SARC028 cohort, where it significantly stratified patients into groups with a median PFS of 170 versus 49 days (HR = 0.71, *p* = 0.007), representing one of the few externally validated transcriptomic biomarkers in sarcoma immunotherapy [9]. Nevertheless, most of the current evidence supporting TLSs and the Sarcoma Immune Classes derives from retrospective analyses of a limited number of cohorts, most notably SARC028, and prospective validation in biomarker-stratified trials is still lacking [71,72,73].

Finally, ecosystem-level biomarkers, including ecotype and SAMS classifications, metabolic signatures, and spatial profiling—represent the most exploratory tier of biomarker development. Rather than measuring isolated molecular events, these approaches attempt to capture the multidimensional interactions between tumor cells, stromal components, and immune populations. Despite their considerable biological appeal, they currently lack harmonized methodologies, a standardized nomenclature, and prospective validation, precluding routine clinical application [47,77,80]. In parallel, peripheral blood-based biomarkers—including circulating T-cell immunotyping, cfDNA derived from active chromatin, and standard hematologic indices—are emerging as complementary, minimally invasive approaches, although they remain at early stages of validation [81,82,83]. Collectively, these observations indicate that future biomarker development in STS should prioritize integrated, subtype-aware models combining tumor-intrinsic features, immune cell composition, spatial organization, antigen presentation competence, and myeloid/stromal contexture.

## 7. Change of Paradigm—Strategies to Enhance Immune Responsiveness in Soft Tissue Sarcoma

**Figure 3:**
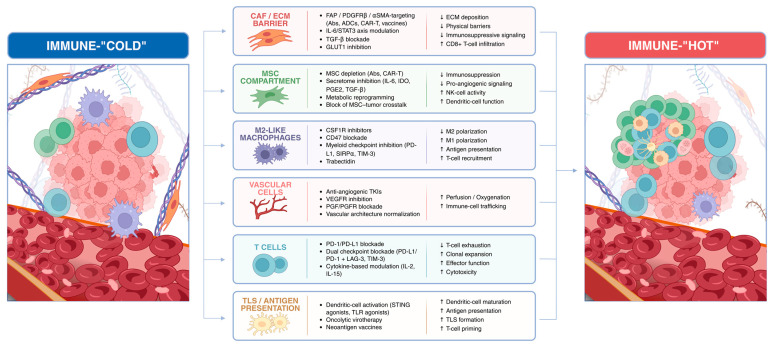
STS microenvironment modulation strategies—Specific cells and microenvironmental compartments/barriers, which are amenable to be modulated, are highlighted αSMA—alpha-smooth muscle actin; Abs—antibodies; ADCs—antibody–drug conjugates; CAR-T—chimeric antigen receptor T-cell; CD—cluster of differentiation; CSF1R—colony-stimulating factor 1 receptor; ECM—extracellular matrix; FAP—fibroblast activation protein; GLUT1—glucose transporter 1; IDO—indoleamine 2,3-dioxygenase; IL—interleukin; LAG-3—lymphocyte activation gene 3; MSC—mesenchymal stromal cell; NK—natural killer; PD-1—programmed cell death protein 1; PD-L1—programmed death-ligand 1; PDGFRβ—platelet-derived growth factor receptor beta; PGE2—prostaglandin E2; PGF—placental growth factor; SIRPα, signal regulatory protein alpha; STING, stimulator of interferon genes; TGF-β, transforming growth factor beta; TIM-3, T-cell immunoglobulin and mucin-domain containing-3; TKIs—tyrosine kinase inhibitors; TLR—Toll-like receptor; TLS—tertiary lymphoid structure; VEGFR—vascular endothelial growth.

Most STSs are intrinsically “immune-cold,” characterized by low TMB, limited CD8^+^ T-cell infiltration, abundant M2-polarized macrophages, and low PD-L1 expression. However, immune resistance is not absolute, and strategies aimed at enhancing tumor immunogenicity, remodeling the TME functional immune profile, or bypassing endogenous priming through adoptive cellular therapies may restore responsiveness to immunotherapy [29,30,32,50] (Figure 3).

### 7.1. Immune Sensitization: From Immune-Based Therapy-Unresponsive to Immune-Based Therapy-Responsive

#### 7.1.1. Editing Intrinsic Tumor Immunogenicity

Strategies aimed at editing intrinsic tumor immunogenicity seek to increase the visibility of sarcoma cells to the adaptive immune system by, for example, enhancing antigen release, antigen presentation, and interferon-driven immune signaling [32].

Firstly, radiotherapy is a well-established modality in this context. By inducing immunogenic cell death, it promotes the release of tumor antigens and damage-associated molecular patterns (DAMPs), enhances MHC class I expression, and activates type I interferon signaling, thereby facilitating dendritic cell priming and CD8^+^ T-cell recruitment. Clinical evidence supporting this priming effect comes from the SU2C-SARC032 trial, where the addition of pembrolizumab to pre-operative radiotherapy improved DFS in high-risk UPS and liposarcoma [12].

Similarly, anthracycline-based chemotherapy (e.g., doxorubicin) induces immunogenic cell death through calreticulin exposure, heat shock protein expression, and HMGB1 release, promoting dendritic cell maturation and antigen presentation to T cells. This leads to activation of tumor-specific IFN-γ-producing T cells and reduced regulatory T-cell induction, fostering a more immunostimulatory microenvironment [84]. Consistently, neoadjuvant anthracycline-based regimens have been associated with augmented immune sensitiveness, improved DFS and pathological response in STS, supporting their immunomodulatory role and rationale for combination with ICIs [85,86]. In addition, DNA-damaging strategies such as PARP inhibition also increase genomic instability and enhance neoantigen generation, particularly in genomically complex subtypes.

Epigenetic modulation also represents a promising approach to enhance tumor immunogenicity, particularly given the frequent chromatin dysregulation observed in specific histotypes such as SS, epithelioid sarcoma, MPNST, rhabdomyosarcoma, and MFS [36]. Agents targeting DNA methylation, histone deacetylation, or EZH2 can reverse the epigenetic repression of immune-related genes, leading to the re-expression of tumor antigens (including CTAs), upregulation of MHC molecules, and restoration of antigen processing machinery. Early studies suggest that combining epigenetic-based therapies with ICIs or adoptive cell therapies may convert immune-cold sarcomas into more inflamed phenotypes and offer interesting ORRs of approximately 10–20%, although optimal patient selection remains under investigation [36,87,88,89].

#### 7.1.2. Broad Tumor Microenvironment Remodeling

Different from tumor-intrinsic strategies, TME remodeling approaches aim to overcome immunosuppressive networks in STS, driven by factors such as abnormal vasculature, hypoxia, and M2-polarized TAMs, that promote T-cell exclusion and impaired antitumor immunity. Therapeutic strategies include anti-angiogenic tyrosine kinase inhibitors (TKIs), trabectedin, dual-checkpoint blockade, macrophage-targeting therapies, and oncolytic virotherapy [90,91,92,93].

##### Anti-Angiogenic TKIs

Anti-angiogenic tyrosine kinase inhibitors remodel the sarcoma’s TME by inhibiting VEGF signaling, modulating microenvironmental vasculature, reducing hypoxia, and improving immune cell trafficking and infiltration. Fundamental and translational studies demonstrate that treatment with different anti-angiogenic TKIs increases intratumoral CD8^+^ T-cell and B-cell infiltration, enhances PD-1 expression, and promotes a shift toward a more inflamed microenvironment, even in immune-cold sarcomas [90,91]. For instance, the REGOMUNE phase II trial combining regorafenib with avelumab showed modest ORRs (11%) but confirmed significant TME remodeling through increased immune cell infiltration and PD-1 upregulation [92].

Consistently, pooled analyses of VEGFR-targeting TKIs combined with ICIs reported ORRs of around 30% and a median PFS of approximately 7 months, exceeding historical monotherapy outcomes. ASPS appears to be particularly sensitive, with ORRs reaching 73%, likely reflecting its VEGF-driven biology [90]. Similar activity has been reported with axitinib–pembrolizumab and cabozantinib-based combinations, supporting vascular and stromal modulation as a relevant immune-sensitization strategy in STS [91,93].

##### Trabectedin

Trabectedin represents a relevant immune-sensitizing strategy in STS through selective depletion of immunosuppressive TAMs, particularly CD68^+^CD163^+^ M2-like populations [94]. Beyond its direct cytotoxic activity, trabectedin reduces pro-inflammatory and pro-angiogenic cytokines such as CCL2 and IL-6, reshaping the TME and indirectly enhancing T-cell permissiveness. These changes correlate with improved PFS and favorable modulation of immune-related gene signatures, increasing the expression of CD3, CD8, perforin, granzyme B, and interferon-responsive genes [95].

Importantly, trabectedin can increase PD-1 expression on T cells and sensitize previously resistant sarcoma models to anti-PD-1 therapy, findings that support the employment of combination strategies with ICIs [94,95,96,97]. These findings highlight that immune sensitization in STS may be achieved through stromal and myeloid reprogramming rather than increased neoantigen generation alone [98].

##### Dual Immune Checkpoint Blockade

Dual immune checkpoint blockade, such as the combination of PD-1/PD-L1 axis inhibitors and CTLA-4 inhibitors, aims to amplify antitumor immunity by simultaneously restoring exhausted effector T cells and enhancing T-cell priming and clonal expansion [11,50]. Different trials have explored the use of combinations such as durvalumab–tremelimumab and nivolumab–ipilimumab, and have demonstrated increased intratumoral CD3^+^, CD8^+^, and PD-1^+^ T-cell infiltration, together with dynamic PD-L1 upregulation during therapy, supporting active immune remodeling within the sarcoma microenvironment [11,99].

Despite providing modest ORRs in unselected populations, this strategy’s activity is enriched in immune-inflamed histologies such as ASPS, UPS, and DDLPS, with ORRs ranging from approximately 10 to 50% depending on subtype [11,50,100]. Importantly, baseline PD-L1 expression alone does not reliably predict benefit, reinforcing the importance of dynamic and microenvironment-based biomarkers.

##### Myeloid Checkpoint Targeting

Given the predominance of immunosuppressive myeloid populations in many STSs, targeting macrophage-related pathways has emerged as another TME-remodeling strategy [101]. Inhibition of CSF1R depletes M2-like TAMs of the TME, reduces regulatory T-cell populations, and enhances CD8^+^ T-cell infiltration, thereby improving tumor control and sensitizing tumors to ICIs and cancer vaccines [101,102,103]. Similarly, CD47 blockade enhances macrophage-mediated phagocytosis and reduces tumor burden in preclinical sarcoma models, although its combination with PD-1 blockade has shown limited additive immune activation [20]. Metabolic immune suppression also contributes to myeloid-mediated resistance. IDO1 promotes tryptophan degradation and accumulation of immunosuppressive kynurenine metabolites, leading to T-cell dysfunction and recruitment of myeloid-derived suppressor cells. Although IDO1 inhibitors have shown limited efficacy as monotherapy, combination approaches with chemotherapy or ICIs remain under investigation [104].

##### SDH Activity Modulation

Succinate dehydrogenase (SDH) enzymatic dysfunction has emerged as a relevant metabolic alteration in STS, particularly in UPS. In this specific histotype, the overexpression of the genes that encode the distinct SDH subunits and the overexpression of oxidative phosphorylation pathways is correlated with poor prognosis [98,105,106]. Despite the overexpression of these SDH subunit-encoding genes, metabolomic analyses point towards an impairment of SDH enzymatic activity, leading to succinate accumulation, which acts as an oncometabolite contributing to epigenetic pro-tumoral modifications. However, succinate has a pleotropic nature, not only promoting epigenetic-based alterations, but also influencing antitumor immunity by modulating antigen presentation pathways and effectiveness and, subsequently, the functional profiles of different immune cells within the TME [107]. Given the prognostic relevance of immune infiltration in UPS and the immunologic effects of succinate accumulation, integrating metabolic profiling with immune stratification may improve patient selection for immunotherapy and support novel combination strategies targeting metabolic and immune pathways simultaneously [108,109,110].

##### Oncolytic Virotherapy

Oncolytic virotherapy represents a distinct TME-remodeling strategy that combines direct tumor lysis with induction of local and systemic antitumor immunity. By promoting immunogenic cell death, oncolytic viruses stimulate release of tumor antigens, DAMPs, and pro-inflammatory cytokines, leading to dendritic cell activation, enhanced antigen presentation, and increased type I interferon signaling [111]. Translational studies consistently demonstrate increased CD8^+^ T-cell infiltration, interferon-response gene expression, and expansion of tumor-specific T-cell clones following treatment with oncolytic virus [112,113].

Clinical studies support these immunologic effects. Combination of talimogene laherparepvec (T-VEC) with pembrolizumab achieved ORRs of 35% in advanced sarcoma, while isolated limb perfusion with T-VEC produced durable responses even in refractory histologies [113,114]. However, other trials, such as METROMAJX, demonstrated that immune activation alone may not be sufficient in profoundly immune-cold tumors, emphasizing the importance of baseline immune competence and effective effector function [111].

#### 7.1.3. Specific Targeting of Microenvironmental Resident Immune Cells

The TME of STS is dominated by resident immune populations that actively regulate immune profiles and immune-based treatment responsiveness. In particular, TAMs are predominantly polarized toward an immunosuppressive M2-like phenotype, characterized by the expression of CSF1R, CD163, CD206, and checkpoint ligands such as PD-L1 and CD47 [64]. These macrophages preferentially localize in perivascular niches, where they promote angiogenesis, suppress antigen presentation, and restrict T-cell infiltration, contributing to poor prognosis and reduced responsiveness to ICIs across several STS histotypes [50,53,64].

Resident T-cell populations also play crucial roles, although their activity is highly context-dependent. While CD8^+^ T cells are frequently present, they often exhibit functional exhaustion associated with inhibitory receptor co-expression, including PD-1, LAG-3, and TIM-3 [32]. Importantly, therapeutic benefit appears to depend less on absolute lymphocyte density than on functional state, spatial organization, and the capacity for reinvigoration following immunotherapy [11,64].

Likewise, B-cell-rich TLSs and dendritic cells contribute to more immune-competent microenvironments associated with improved survival and responsiveness to ICIs and increased survival, while NK cells may provide complementary antitumor activity, particularly in tumors with defective antigen presentation [30,32,53,65].

Overall, current evidence suggests that therapeutic responsiveness in STS is determined not only by baseline immune composition, but also by the dynamic capacity of resident immune populations to undergo functional reprogramming during treatment. Consequently, macrophages, resident T cells, B-cell-rich TLSs, and antigen-presenting cells are increasingly being regarded as active therapeutic targets capable of reshaping immune-cold sarcomas into more responsive microenvironments [11,64,65,85].

#### 7.1.4. Specific Targeting of Microenvironmental Resident Non-Immune/Stromal Cells

Beyond immune cells, non-immune stromal compartments in STS, particularly CAFs, mesenchymal stromal cells (MSCs), endothelial cells and pericytes, actively shape immune exclusion by a panoply of mechanisms, vascular dysfunction, and therapy resistance, and are therefore increasingly viewed as valuable actionable targets to enhance immune responsiveness rather than simply passive bystanders. Indeed, these stromal programs can restrict cytotoxic lymphocyte access, dampen antigen-driven effector function, and create metabolic and cytokine conditions that favor immune suppression, implying that successful immunotherapy in STS will often require co-targeting stromal barriers that prevent immune engagement [53,115,116,117].

CAFs are a dominant stromal population in many STSs and display substantial functional heterogeneity, with specific CAF states exerting disproportionate effects on immune trafficking and drug response. Mechanistic studies have identified a glycolytic-enriched CAF subset (glyCAF), whose density is increased at the invasive margin of immune-excluded STSs. Consistently, these CXCL16^+^ glyCAFs form a “barrier” that spatially separates CD8^+^ T cells from malignant cells and limits penetration into the tumor parenchyma. Importantly, immune exclusion appears to be driven less by CAF abundance than by CAF subtype and localization at the tumor margin. The inhibition of CAF glucose uptake via GLUT1 blockade reduces CXCL16 expression in glyCAFs and increases intratumoral CD8^+^ T-cell infiltration. Moreover, the combination of GLUT1 inhibition with doxorubicin reduced tumor growth in a T-cell-dependent manner, supporting stromal metabolic targeting as an immune-sensitizing strategy. More broadly, CAFs promote immune escape and resistance through extracellular matrix remodeling, secretion of immunosuppressive cytokines, and checkpoint-relevant crosstalk (e.g., LGALS9/TIM-3), motivating strategies that include CAF depletion, functional reprogramming toward less suppressive states, metabolic targeting, and disruption of stromal–tumor–immune signaling loops [53,115,118].

In parallel, several CAF-directed therapeutic paradigms are being explored across different cancer types and are increasingly shaping sarcoma trial design. Firstly, direct CAF targeting/depletion via the inhibition of specific surface markers such as FAP, PDGFRβ and αSMA is being tested using antibodies, antibody–drug conjugates (ADCs), CAR-T approaches, vaccines, and radiopharmaceuticals. Secondly, blocking CAF–tumor/immune crosstalk (notably inflammatory cytokine circuits such as IL-6/STAT3 and chemokine axes that compartmentalize T cells) is also being attempted. Lastly, reestablishing the “normalization” of CAF functional programs (e.g., inhibiting TGF-β-linked fibroblast activation rather than indiscriminate ablation) is another compelling strategy. Collectively, these concepts support combination strategies in which stromal interference is used to “open” the tumor microenvironment to immune infiltration and then to couple it with ICIs or other types of immune-based therapy [53,118,119].

Similarly, MSCs represent an additional, highly plastic non-immune stromal cell type, implicated both as a candidate cell of origin of some specific sarcoma histotypes and also as an active microenvironmental modulator once “educated” by tumor-derived signals. Notably, in pro-inflammatory or cytokine-rich environments (e.g., TGF-β, IFN-γ, and VEGF), MSCs commonly display tumor-supportive functional phenotypes, promoting angiogenesis, immune evasion, invasion and treatment resistance. Mechanistically, MSCs (and MSC-derived extracellular vesicles) can convey immunosuppressive mediators such as IL-6, IDO, PGE2 and TGF-β, suppressing dendritic cell antigen presentation, impairing NK cell differentiation/function, and favoring regulatory and myeloid-skewed programs. Thus, different therapeutic approaches include the modulation of pro-tumoral MSC niches, inhibiting their immunosuppressive secretome, reprogramming its metabolic profiles, and blocking the crosstalk with sarcoma cells. MSC-targeting approaches should be framed by careful safety consideration given the documented tumor-promoting potential MSCs show in certain contexts, being attractive for tumor-tropic delivery of cytokines, interferons, TRAIL, anti-angiogenics or even oncolytic payloads [116,117,120,121].

In addition, the vascular–perivascular stromal axis is also potentially actionable. Endothelial functional profiles associated with immune-depleted microenvironments, illustrated by PGF^+^ endothelial tip-cell enrichment, are correlated with poor prognosis and may contribute to dysfunctional vasculature networks, hypoxic microenvironmental landscapes and impaired immune trafficking. Likewise, pericyte subsets (including fibrogenic pericytes) participate in extracellular matrix production, shape extracellular matrix composition, and promote a vast array of vascular architectural abnormalities that reinforce immune exclusion. Consequently, targeting tumor-associated endothelial cells and pericytes is framed as a strategy to improve immune cell access, potentially converting physical exclusion into immune-permissive conditions that enable downstream immunotherapy efficacy [53,122,123].

Finally, additional stromal features may contribute to immune escape and therapeutic resistance in selected contexts. As an example, cancer-associated adipocytes at invasive fronts can support matrix remodeling, invasion and inflammatory cytokine production, and may also fuel tumor metabolism through lipolysis-driven substrate transfer. Overall, these observations reinforce a central premise of the paradigm shift in sarcoma immuno-oncology: enhancing immune responsiveness often requires dismantling stromal and vascular barriers. Through CAF/MSC reprogramming, vascular normalization, and blockade of stromal crosstalk, immune effector mechanisms are rendered functionally relevant in STS [53,118,119,122].

### 7.2. Immunomodulatory Effects of Approved Drugs on the Tumor Microenvironment

Conventional therapies in STS are increasingly recognized as active modulators of the TME rather than purely cytotoxic agents. Radiotherapy, chemotherapy, and targeted therapies can reshape antigen presentation dynamics, immune cell infiltration profiles, and suppressive networks, thereby conditioning responsiveness to ICIs [50,53]. Importantly, immune modulation is often dynamic and treatment-induced, as clinically relevant changes in immune activation and checkpoint expression frequently emerge during therapy rather than at baseline [11]. Radiotherapy and anthracycline-based chemotherapy promote immunogenic cell death, antigen release, dendritic cell priming, and T-cell recruitment, although compensatory immunosuppressive pathways such as myeloid activation and IDO1 signaling may also arise [32,53,124].

Similarly, anti-angiogenic TKIs improve immune accessibility through vascular architecture alteration and hypoxia gradient modulation, while agents such as trabectedin counteract myeloid-driven immune suppression by modulating the activity of TAMs [30,66]. Emerging approaches such as nanomedicine may further enhance this immune-sensitizing effect by optimizing drug delivery and simultaneously overcoming stromal and vascular barriers, thereby facilitating CD8^+^ T-cell infiltration and improving responsiveness to PD-L1 blockade [30,125]. Collectively, these findings support a paradigm in which conventional sarcoma therapies are repurposed as TME-modifying platforms designed to sensitize immune-cold tumors to immunotherapy [11,50,66].

## 8. Therapeutic Armamentarium in Soft Tissue Sarcomas

### 8.1. Immune Checkpoint Inhibitor Monotherapy: The Paradigmatic Case of Atezolizumab in Advanced Alveolar Soft Part Sarcoma

ASPS represents a striking exception within the generally immune-refractory landscape of STS, demonstrating consistent and durable sensitivity to ICI monotherapy [93]. Consequently, ASPS has emerged as a paradigmatic example of histology-driven immunotherapy in sarcomas, where single-agent PD-1/PD-L1 blockade can produce meaningful and long-lasting clinical benefit [126,127,128,129].

A phase III trial evaluated the clinical benefit of treatment with atezolizumab in monotherapy in adult and pediatric patients with unresectable or metastatic ASPS. Treatment with atezolizumab achieved an ORR of 37%, with durable partial and complete responses, a median PFS of 20.8 months, and a median duration of response exceeding 24 months [127]. These findings corroborated earlier phase trial data, which had reported response rates approaching 40–45% in heavily pretreated patients [93]. Importantly, responses were often delayed but sustained, with evidence of prolonged disease control even after treatment interruption. Moreover, pseudo-progression, characterized by dense lymphocytic infiltration and non-viable tumor tissue, has also been documented, supporting a genuine immune-mediated mechanism of response [127,129].

Notably, the clinical efficacy of atezolizumab occurs despite biological features traditionally associated with resistance to immunotherapy, including low TMB and variable baseline PD-L1 expression [127,128,129]. Instead, response appears to depend primarily on immune contexture and treatment-induced immune remodeling. Atezolizumab has been shown to increase intratumoral CD8^+^ cytotoxic T cells, enhance TCR signaling, improve effector-to-regulatory T-cell ratios, and dynamically upregulate PD-L1 expression during therapy, with several initially non-inflamed tumors converting to immune-responsive phenotypes [127]. In particular, the presence of intratumoral CD8^+^PD-1^+^ T cells appears more predictive of response than PD-L1 expression or genomic alterations alone [128].

Collectively, ASPS establishes a benchmark for successful ICI monotherapy in STS and challenges the assumption that a high TMB or high immune checkpoint receptor expression is required for immunotherapy efficacy. Instead, it demonstrates that, in biologically permissive contexts potentially driven by the ASPSCR1–TFE3 fusion and preserved immune trafficking, PD-L1 blockade alone can achieve transformative and durable clinical benefit [93,126,127,129].

### 8.2. Immune Checkpoint Inhibitor Combinations with Local Treatment Modalities

Because the efficacy of ICI monotherapy remains limited in most STSs, combinations with local therapies have emerged as a strategy to enhance antitumor immunity and overcome immune resistance [130]. Radiotherapy is particularly relevant in this context, as it induces immunogenic cell death, promotes tumor antigen release, enhances MHC class I expression, activates type I interferon signaling, and facilitates dendritic cell maturation and CD8^+^ T-cell priming [29,130]. At the same time, radiotherapy may induce adaptive resistance mechanisms, including PD-L1 upregulation and recruitment of a panoply of immune-permissive myeloid populations, thereby providing a rationale for combination with ICIs [131]. Clinically, the SU2C-SARC032 trial demonstrated that adding perioperative pembrolizumab to standard neoadjuvant radiotherapy and surgery improved disease-free survival in patients with high-risk localized UPS and DDLPS, supporting the concept that radiotherapy-induced immune priming can translate into durable clinical benefit [12].

Beyond radiotherapy, oncolytic viruses have also shown immune-sensitizing potential. For instance, T-VEC promotes direct tumor lysis together with local innate and adaptive immune activation, and, when combined with pembrolizumab, it achieved ORRs of around 35% in advanced sarcomas, accompanied by increased tumor-infiltrating lymphocytes and immune-related gene expression [29,32]. More recently, next-generation viral platforms such as AdAPT-001 and OH2 have demonstrated encouraging activity, including reversal of prior ICI resistance, increased CD8^+^ T-cell infiltration, and occasional abscopal responses, reinforcing the role of local immune activation in reshaping the sarcoma TME [132,133]. Additional strategies combining radiotherapy with intralesional immunostimulatory agents, including STING agonists, Toll-like receptor agonists, CD40 agonists, and cytokines, further aim to amplify local immune priming while limiting systemic toxicity [134].

Collectively, these approaches support the concept that local therapies can function as immune-sensitizing platforms capable of converting immune-cold sarcomas into more immune-responsive states [12,131]. However, unlike atezolizumab monotherapy in ASPS, none of these combination strategies are currently approved as standard-of-care therapeutic approaches in STS, and their role remains investigational despite encouraging translational and clinical results.

### 8.3. Immune Checkpoint Inhibitor Combinations with Systemic Treatment Modalities

Given the limited efficacy of ICI monotherapy in STS, combinatory strategies employing ICI and systemic therapies have emerged as important approaches to enhance immune responsiveness and overcome intrinsic resistance by modulating antigen presentation, vascular architecture, and immunosuppressive cell populations within the TME [135]. A meta-analysis including 27 trials has shown that combinations of anti-PD-1/PD-L1 agents with chemotherapy achieved pooled ORRs of approximately 20%, reaching 23% in the first-line setting. Regimens such as pembrolizumab plus doxorubicin and nivolumab–ipilimumab combined with trabectedin demonstrated improved disease control and survival outcomes compared with historical chemotherapy benchmarks, supporting the role of cytotoxic agents as immune-sensitizing partners [135]. Similarly, combinations with VEGF-directed TKIs produced ORRs of approximately 20–24%, with notable activity in ASPS and UPS. Notably, pembrolizumab combined with axitinib achieved a 3-month PFS rate of 65.6% in advanced sarcomas [93].

Dual immune checkpoint blockade has also demonstrated enhanced activity compared with ICI monotherapy. In the Alliance A091401 trial, nivolumab plus ipilimumab achieved a confirmed ORR of 16%, compared with 5% for nivolumab alone, supporting the role of CTLA-4 inhibition in augmenting PD-1 blockade through enhanced T-cell activation and expansion [136]. Likewise, durvalumab combined with tremelimumab showed histology-specific benefit, particularly in ASPS, where ORRs reached 40–50% and early PFS exceeded 80% [11]. Importantly, combinations integrating immunogenic chemotherapy with checkpoint blockade may further broaden responsiveness in traditionally immune-cold sarcomas. For example, doxorubicin combined with balstilimab and zalifrelimab achieved an ORR of 33.3% and a disease control rate of 80% across multiple subtypes [86]. Collectively, these findings support a shift away from isolated ICI monotherapy toward mechanism-driven combination strategies tailored to tumor biology and immune contexture in STS [86,135,136].

### 8.4. Adoptive Cellular Therapies: Engineered T-Cell Approaches

One of the most transformative immunotherapeutic strategies in sarcomas emerged from adoptive cellular therapy using genetically engineered T cells. Given that immune checkpoint inhibition is generally conditioned by low TMB, neoantigen diversity, and immunosuppressive tumor microenvironments, these strategies aim to address these constraints and overcome them [30]. Unlike ICIs, which rely on pre-existing antitumor immunity, engineered T-cell therapies bypass defective endogenous priming by supplying large numbers of tumor-specific lymphocytes with predefined antigen specificity [137].

**Figure 4:**
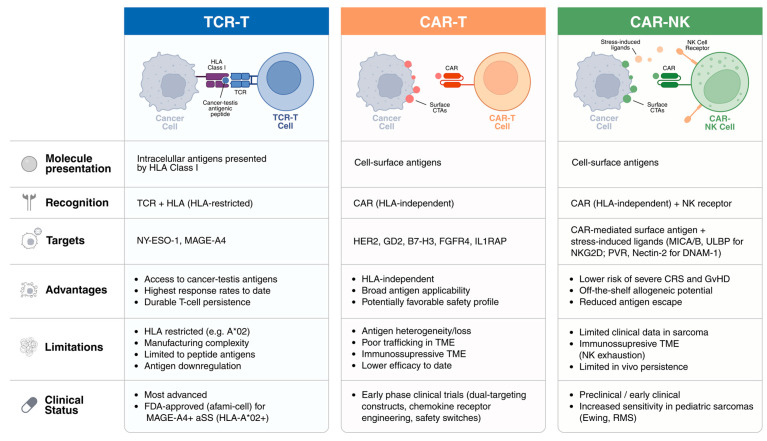
Adoptive cellular therapy platforms for STS. aSS—advanced synovial sarcoma; CAR—chimeric antigen receptor; CAR-T—chimeric antigen receptor T cell; CRS—cytokine release syndrome; CTA—cancer–testis antigen; GvHD—graft versus host; HLA—human leucocyte antigen; RMS—rhabdomyosarcoma; TCR-T—T cell receptor-engineered T cells; TME—tumor microenvironment.

There are two major platforms that dominate ACT development in sarcomas: T-cell receptor-engineered T cells (TCR-T) and chimeric antigen receptor T cells (CAR-T), with CAR-NK also showing promising data (Figure 4). Up to now, TCR-T therapies have demonstrated the most compelling and reproducible clinical efficacy [138]. Notably, these approaches exploit the frequent expression of CTAs, such as NY-ESO-1 and MAGE-A4, in selected translocation-driven sarcomas, particularly SS and MRCL, enabling targeting of intracellular antigens presented by HLA class I molecules [139].

The most robust clinical validation of this strategy is provided by the SPEARHEAD-1 phase II trial, where afamitresgene autoleucel (afami-cel), an affinity-enhanced MAGE-A4-specific TCR-T product, achieved an ORR of 37% in heavily pre-treated HLA-A*02-positive patients with advanced SS and MRCL, with durable responses and manageable toxicity [140]. Remarkably, clinical benefit was observed in tumors refractory to immune checkpoint blockade and correlated with antigen expression levels and long-term persistence of infused T cells, highlighting both antigen dependency and cellular durability as key determinants of efficacy and culminating in the first FDA approval of an engineered T-cell therapy for sarcomas (specifically for HLA-A*02-positive patients with advanced synovial sarcomas that express MAGE-A4) [141].

In parallel, the employment of an NY-ESO-1-targeted TCR-T therapy (Letetresegene Autoleucel (Lete-Cel)) (for HLA-A*02-positive patients) has produced impressive ORRs in synovial sarcoma and MRCL in the ongoing phase II IGNYTE-ESO trial, reinforcing CTA-targeted TCR therapy as the most mature ACT modality in sarcomas and positioning TCR therapies as precision immunotherapies capable of overcoming immune ignorance when a stable, tumor-restricted antigen is present [30,138]. Indeed, Lete-Cel demonstrated an ORR of 61% in HLA-A*02:01, -*02:05, or -*02:06-positive patients, with NY-ESO-1 expressing advanced SS in earlier clinical trials, showing durable tumor regression and estimated 3- and 5-year survival rates of 38% and 14%, respectively [142,143]. Furthermore, subsequent biomarker analyses confirmed an ORR of approximately 50%, with clinical benefit correlating with higher effector-memory CD8^+^ T-cell doses, cytokine release signatures, and robust in vivo T-cell expansion [142]. Similarly, it has been reported that MRCL achieved ORRs of 20–40% following treatment with Lete-Cell, with the magnitude of clinical benefit depending on lymphodepletion intensity, supporting the relevance of conditioning regimens for optimal cellular expansion and persistence [144]. Additionally, other NY-ESO-1-specific TCR-T platforms, including TBI-1301 and TAEST16001, have demonstrated ORRs of approximately 40–50% in SS, with manageable cytokine release syndrome and encouraging durability of response [145]. Lastly, experimental strategies combining NY-ESO-1-specific TCR-T cells with dendritic cell vaccination, nanoparticle peptide vaccines, and hematopoietic stem cell co-infusion are also under investigation to enhance T-cell persistence and overcome immune suppression [146,147,148].

Conversely, CAR-T cell therapies have demonstrated more limited efficacy in sarcomas [139]. Although CAR-T cells offer the advantage of HLA-independent antigen recognition, their clinical activity has been constrained by the scarcity of truly sarcoma-specific surface antigens, heterogeneous antigen expression, impaired trafficking through dense stroma, and profound immunosuppression within the sarcoma TME [141]. Nonetheless, early-phase trials evaluated different types of CAR-T cell therapies targeting antigens such as HER2, GD2, B7-H3, FGFR4, and IL1RAP. Although technical feasibility and acceptable safety profiles have been shown, profound and durable response rates were infrequent [139]. To address these limitations, ongoing efforts are focusing on dual-targeting constructs, chemokine receptor engineering, safety switches, and alternative cellular platforms, including CAR-NK cells, which may mitigate exhaustion and off-tumor toxicity [137].

Despite the somewhat promising premises, engineered T-cell therapies face important challenges that currently limit broader applicability, including HLA restriction (predominantly A*02), strict antigen expression thresholds, manufacturing complexity, lymphodepleting chemotherapy-related toxicity, and the existence of prototypical hostile TMEs characterized by suppressive myeloid populations, regulatory T cells, metabolic stress, and physical barriers to infiltration [137,138]. Consequently, future progress will depend on overcoming HLA restriction, expanding the antigenic repertoire, improving T-cell persistence and trafficking, refining conditioning regimens, and integrating ACT with complementary strategies such as immune checkpoint blockade, radiotherapy, or microenvironment-modulating agents [30,141].

Beyond efficacy, the safety profile of engineered T-cell therapies is a critical determinant of their clinical applicability.

Direct evidence regarding the safety of TCR-T and CAR-T therapies in sarcoma-specific cohorts remains limited, with most current recommendations being extrapolated from the substantially larger experience in hematologic malignancies [83,149]. Consequently, although the overall toxicity profile is considered comparable, the incidence and severity of adverse events in STS remain incompletely characterized and should be interpreted cautiously [150].

The most frequently reported toxicities include cytokine release syndrome (CRS) and immune effector cell-associated neurotoxicity syndrome (ICANS).

CRS driven by supraphysiologic cytokine release (IL-6, IFN-γ) upon T-cell activation and expansion is the most frequent toxicity associated with both TCR-T and CAR-T platforms and is graded according to the ASTCT consensus criteria (grade 1–4, based on fever, hypotension, and hypoxia) [151,152]. Sarcoma-specific data are now available for TCR-T therapy: in the pivotal SPEARHEAD-1 trial, CRS occurred in the majority (71%) of patients but was predominantly low-grade (grade 1–2), manageable with supportive care and, when required, with tocilizumab and/or corticosteroids, rarely leading to treatment discontinuation. Indeed, grade ≥3 was verified in only 2% of patients, and no treatment-related deaths have been reported [140]. Similar low-grade, manageable CRS has been reported with lete-cel in the IGNYTE-ESO trial and with other NY-ESO-1-directed TCR-T platforms (TBI-1301 and TAEST16001) [145,146].

ICANS, also graded using the ASTCT ICE score, was likewise rare in the SPEARHEAD-1 trial, occurring in only one patient (2%, grade 1) [131].

These figures contrast favorably with the CAR-T experience in hematologic malignancies, where grade ≥3 CRS and ICANS rates are substantially higher [83,149], likely reflecting the more physiological signaling of TCRs through the endogenous CD3 complex compared with the synthetic costimulatory domains of CARs [153,154]. 

Conceptually, current management is based on supportive care, while corticosteroids and tocilizumab are reserved for persistent or severe CRS [149]. Conversely, ICANS is managed primarily with corticosteroids, together with intensive neurological monitoring and supportive care, as IL-6 blockade alone has limited efficacy in isolated neurotoxicity [149,155].

Additional clinically relevant toxicities include prolonged cytopenias—which represent the predominant toxicity burden with TCR-T therapy in sarcomas, with grade ≥3 lymphopenia in 96% and neutropenia in 85% of patients in SPEARHEAD-1—infectious complications following lymphodepleting chemotherapy, hemophagocytic lymphohistiocytosis (HLH)-like syndromes, and on-target/off-tumor toxicity, the latter being of particular concern in solid tumors because many target antigens are also expressed, at lower levels, in normal tissues [131,139,145]. The clinical significance of this risk was patent in early trials of affinity-enhanced TCRs targeting MAGE-A3, where fatal cardiovascular toxicity resulted from cross-reactivity with titin and lethal neurotoxicity from cross-reactivity with MAGE-A12 expressed in the brain [156,157]; these events prompted a fundamental redesign of preclinical screening protocols, and the TCRs currently in clinical use—afamitresgene autoleucel (MAGE-A4) and letetresgene autoleucel (NY-ESO-1)—have not demonstrated clinically significant off-tumor toxicity [131,158].

Early-phase CAR-T trials in sarcoma targeting HER2, GD2, B7-H3, FGFR4, and IL1RAP and CAR-NK have similarly reported acceptable and manageable safety profiles, without unexpected on-target/off-tumor toxicity, although experience remains more limited than for TCR-T platforms.

Overall, the available evidence indicates that TCR-T therapy has been more extensively evaluated than CAR-T and CAR-NK in sarcomas, particularly in SS, and demonstrates a more well-documented acute toxicity profile characterized by predominantly low-grade CRS and rare ICANS, whereas CAR-T and CAR-NK experience remains limited and safety algorithms continue to rely largely on evidence generated in hematologic malignancies [83,149,155]. Therefore, future prospective sarcoma-specific studies are needed to better define toxicity incidence, optimize management strategies, and improve the therapeutic index of engineered T-cell therapies in STS [150,159].

Table 3 resumes the main adoptive cellular therapies for sarcomas, the most relevant safety concerns for each one of them, the conceptual management principles and additional interpretative notes.

In summary, adoptive cellular therapies, such as CTA-specific TCR-engineered T cells, represent a paradigm-shifting advance in sarcoma treatment. While currently they are only applicable to a well-defined subset of sarcomas, their success provides evidence that durable, antigen-directed immune control is achievable in solid tumors [140]. In the same way, continued innovation in cell engineering and rational combination strategies are expected to expand the therapeutic reach of ACT and establish it as a key strategy in personalized sarcoma immunotherapy [137,139].

### 8.5. Neoadjuvant Immunotherapy: Robust Evidence

The randomized SU2C-SARC032 trial provides robust clinical evidence supporting the use of neoadjuvant immunotherapy in STS. In patients with resectable high-risk stage III UPS or DDLPS, the addition of pembrolizumab to standard preoperative radiotherapy and surgery resulted in a significant improvement in DFS, with a 15% increase in 2-year DFS compared with radiotherapy and surgery alone (67% vs. 52%; HR 0.61) at a median follow-up of 43 months [12]. The benefit was particularly pronounced in grade 3 STSs, supporting the notion that highly aggressive sarcomas may be especially susceptible to immune modulation in the neoadjuvant setting. Importantly, concurrent pembrolizumab and radiotherapy demonstrated acceptable toxicity without increased surgical complications [12].

Neoadjuvant studies have also highlighted that immune-mediated pathological responses may occur despite limited radiographic tumor shrinkage, underscoring the limitations of the RECIST criteria and supporting the value of pathological and immunologic assessment in sarcomas [32]. In parallel, tumors with pre-existing immune infiltration, such as SIC E sarcomas, appear to derive greater benefit from PD-1 blockade, reinforcing the rationale for earlier therapeutic intervention even in low-TMB tumors [50]. Moreover, neoadjuvant trial designs uniquely allow paired pre- and post-treatment tumor analyses, enabling direct evaluation of immune remodeling, resistance mechanisms, and biomarker discovery [50]. Collectively, these findings support neoadjuvant immunotherapy as a promising strategy for immune priming and biomarker-driven patient stratification in STS [32].

## 9. Future Perspectives

The future of immunotherapy in STS will rely on refined molecular-based patient selection and precision immune-oncology approaches, in contrast to broad and agnostic immune-flavored applications [30].

The limited efficacy of ICIs in unselected STS largely reflects their intrinsic molecular and immune heterogeneity and their microenvironmental diversity, predominantly populated by dominant immunosuppressive cell populations, and the shortage of reliable predictive biomarkers, while simultaneously highlighting opportunities for biomarker-driven stratification and rational therapeutic design [52,53].

### 9.1. Biomarker-Driven Selection as the Central Hope

There is a major future opportunity based on refined patient selection using biologically meaningful immune biomarkers, whose use should be framed by biological contexts and which should be employed in combination with other different biomarkers, rather than using PD-L1 or TMB alone [124]. Hence, besides these classical immune features, TLS and B-cell-rich immune ecosystems repeatedly emerge as robust predictors of PD-1 blockade benefit [32]. Similarly, molecular type-aware selection has been reinforced since there are certain subtypes such as ASPS, and SMARCA4-deficient tumors that show reproducible immunotherapy sensitivity [73]. The quest for additional immune-sensitivity biomarkers is still in its preliminary stages.

### 9.2. Combinations as Opposed to Monotherapy

The shift away from ICI monotherapy toward rational combination strategies designed to overcome immune exclusion and myeloid dominance is another shared future direction [53]. The need to combine ICIs with chemotherapy, antiangiogenic agents, radiotherapy, oncolytic viruses, and other immune-based modulators of TMEs has been underlined since it provenly leads to greater clinical benefit, while enhancing antigenicity, normalizing vasculature, and reconditioning suppressive TMEs [124]. As previously mentioned, besides combinations of ICI with different treatment classes, there will also be a preponderant role for combinations between different types of immune-based strategies, whose mechanisms may be symbiotic for anti-neoplastic purposes. Nonetheless, there is an important caution that is important to consider: not all combinations were proven to be beneficial, and some may even be counterproductive, highlighting the need for biology-driven rather than empiric combination design [73].

### 9.3. Replacement of the Scope Towards the Microenvironment

As highlighted throughout the work, microenvironmental modulation is emerging as a central paradigm in STS immunotherapy, reflecting a fundamental shift in understanding the determinants of therapeutic response. In contrast to more immunogenic malignancies, tumor-intrinsic features such as TMB, neoantigen load, and PD-L1 expression are inconsistently present and fail to reliably predict therapeutic response, reflecting the intrinsic biological limitations of sarcoma cells as immunologic targets [15,18].

Conversely, the TME has emerged as the dominant regulator of immune responsiveness in STS. Across multiple studies, immune-enriched TMEs consistently outperform tumor-intrinsic biomarkers in predicting response to immune checkpoint blockade [32,60]. Accordingly, therapeutic strategies are increasingly directed toward reshaping this hostile ecosystem. Approaches such as vascular normalization, myeloid cell reprogramming, and induction of immune-permissive niches (for example, through metabolic modulation) have demonstrated the capacity to restore immune infiltration and potentiate checkpoint blockade, highlighting the TME as a more tractable and broadly applicable target across the highly heterogeneous sarcoma subtypes [53,93,125]. In essence, while tumor-intrinsic immunogenicity in STS seems to be hard to tackle, the surrounding microenvironment remains plastic, dynamic and modifiable. Thus, the future of immunotherapy in sarcomas lies not only in attempting to modify the intrinsic immunogenicity of the tumor cell, but also in reprogramming the context in which it exists, transforming immune-resistant landscapes into environments capable of sustaining effective and durable antitumor responses.

### 9.4. Antigen-Directed Cellular Therapy as a Major Source of Optimism

There is compelling evidence supporting the role of adoptive cellular therapies, particularly engineered TCR therapies targeting CTAs (NY-ESO-1 and MAGE-A4) and other fusion-associated targets, as promising routes to bypass the low neoantigen burden that limits checkpoint blockade in many STSs [30,50]. However, the potential success rate remains constrained by HLA restriction, antigen heterogeneity, manufacturing complexity, toxicity management, and limited patient eligibility, which collectively represent constraints on the scalability of ACT approaches [29,30].

### 9.5. Recognizing and Overcoming Biophysical and Clinical Barriers to the Use of Immunotherapy to Treat Soft Tissue Sarcomas

The biophysical nature of mesenchymal tissues, the protypical deep anatomical location of STSs, and the structural and chemical constraints of their TME collectively impose profound pharmacokinetic and pharmacodynamic challenges for immune-based therapy development. Unlike epithelial tumors, STSs arise within connective tissue compartments characterized by heterogeneous vascularization and dense extracellular matrix (ECM), often in anatomically deep sites such as the retroperitoneum or pelvis, where large tumor volumes and limited accessibility further hinder effective drug delivery [160,161]. At the tissue level, sarcoma cells actively produce a rigid and complex ECM enriched in collagen, glycosaminoglycans, and subtype-specific stromal networks, which not only restrict macromolecule diffusion but also directly impair immune cell function and trafficking [157,162]. These barriers are exacerbated by elevated interstitial fluid pressure, which abolishes convective transport and creates pharmacologic “dead zones” within the tumor core, as well as by abnormal, poorly organized vasculature that results in heterogeneous perfusion and limited drug penetration [160,162,163]. Additionally, hypoxia and metabolic acidosis further suppress immune effector function and antigen presentation, reinforcing an environment that is both physically and biologically resistant to immunotherapy [164,165,166,167,168,169,170]. These multiscale physical barriers are even more pronounced for cellular therapies, which must overcome constraints in extravasation, matrix navigation, and metabolic fitness within this hostile milieu [171,172]. Altogether, these features define STSs as tumors in which therapeutic failure is not only a consequence of immune resistance, but also of biophysical inaccessibility, underscoring the need for strategies that simultaneously address drug delivery, stromal architecture, and microenvironmental conditioning to achieve effective antitumor responses [125].

Notably, the development and validation of immune-based therapies in STSs are further exacerbated by the intrinsic difficulties of conducting clinical trials in such rare and heterogeneous malignancies. The fact that STSs are subdivided into more than 100 distinct histological entities creates a fundamental methodological paradox: trials broad enough to ensure feasibility dilute histotype-specific therapeutic signals, whereas biologically refined studies remain underpowered and often infeasible. Consequently, most available evidence arises from small, frequently single-arm phase II trials, with limited capacity to establish definitive efficacy [11,126,135,173]. Early-phase studies are additionally constrained by the need for repeated biopsies in deep-seated tumors and by the absence of reliable pharmacodynamic markers across heterogeneous microenvironments [32,54]. In later phases, conventional randomized trial designs become nearly unattainable, requiring international collaboration, statistical compromises, and alternative methodologies such as basket trials or Bayesian frameworks [174,175]. These challenges are further compounded by immunotherapy-specific factors, including delayed response kinetics, the need for biomarker-driven selection, and the complexity of combination strategies, all of which increase trial duration and design complexity [126,135].

### 9.6. Key Challenges That Define the Roadmap

Despite all the supporting evidence on these more recent approaches, there are several persistent barriers remaining, such as dominant immunosuppressive macrophage programs and stromal exclusion, limited universal biomarkers, inconsistent performance of PD-L1/TMB, trial design limitations in rare diseases, and endpoint challenges, including the inadequacy of RECIST to fully capture immune-mediated benefit [32]. There is, however, an additional strategic perspective which relies on considering the timing of application of different immune-based therapies as a crucial factor since advanced tumors often exhibit substantial, dynamic and clonal-pressure-dependent immunoediting. Thus, perioperative and neoadjuvant designs may provide more favorable contexts for immune engagement and biomarker discovery [29]

## 10. Conclusions: Fiction or Reality?

At first glance, immunotherapy in STS appears to exist between promise and uncertainty. Despite the transformative success of ICIs in several solid tumors, their efficacy in STS has remained limited, largely due to low TMB, the rarity of MSI, limited actionable antigens, extensive inter and intratumoral heterogeneity, and the predominance of immune-cold microenvironments enriched in immunosuppressive myeloid populations [16,32]. Nevertheless, growing evidence indicates that responsiveness to immunotherapy is strongly determined by tumor immune contexture, supporting biomarker-driven patient stratification as a central principle for future therapeutic development. In particular, TLSs and B-cell-rich immune ecosystems have emerged as robust predictors of response to PD-1 blockade, outperforming classical biomarkers such as PD-L1 expression and TMB alone [32,124].

Importantly, ASPS represents a paradigmatic exception within the generally immune-refractory sarcoma landscape. Atezolizumab has demonstrated impressive ORRs and prolonged PFS in clinical trials enrolling patients with ASPS, despite a low TMB and variable PD-L1 expression [93,127,129]. These findings suggest that immunotherapy efficacy in sarcomas may depend less on traditional genomic biomarkers and more on dynamic remodeling of the TME, including expansion of intratumoral CD8^+^ T cells and restoration of effective immune signaling [127,128]. Consequently, ASPS has become a landmark example demonstrating that durable immunotherapy responses in sarcomas are achievable in biologically permissive contexts.

Perhaps the most transformative conceptual advance to emerge from recent research is the recognition that immune resistance in STS may be reversible. Contemporary strategies increasingly aim to convert immune-cold tumors into immune-responsive states by either enhancing intrinsic tumor immunogenicity or by remodeling the immunosuppressive tumor microenvironment through radiotherapy, chemotherapy, epigenetic modulation, anti-angiogenic therapies, macrophage-targeting approaches, oncolytic virotherapy, or rational immune-based combinations [32,36,53,92]. In parallel, adoptive cellular therapies targeting CTAs such as NY-ESO-1 and MAGE-A4 may bypass the low neoantigen burden characteristic of many sarcomas [29,30]. Thus, immunotherapy in STS is progressively evolving from an aspirational concept into a biomarker-guided and increasingly biologically plausible therapeutic reality.

## Figures and Tables

**Table 1:** Histotype-specific expected immunotherapy sensitivity, key immune and molecular characteristics, current evidence supporting immunotherapy use, and future directions for immune-based therapeutic strategies across soft tissue sarcoma (STS) histotypes. Histotypes are ranked according to the current understanding of their relative responsiveness to immunotherapy, integrating tumor-intrinsic biology, immune microenvironment, and available clinical evidence. ACT, adoptive cell therapy; AYAs, adolescents and young adults; ASPS, alveolar soft part sarcoma; CTA, cancer–testis antigen; CD8^+^, cluster of differentiation 8-positive cytotoxic T lymphocyte; DDLPS, dedifferentiated liposarcoma; EED, embryonic ectoderm development; HLA, human leukocyte antigen; HLA-A*02, human leukocyte antigen A02 allele; ICI, immune checkpoint inhibitor; LMS, leiomyosarcoma; MAGE-A4, melanoma-associated antigen A4; MFS, myxofibrosarcoma; MMR, mismatch repair; MPNST, malignant peripheral nerve sheath tumor; MRCL, myxoid round cell liposarcoma; MSI, microsatellite instability; NY-ESO-1, New York esophageal squamous cell carcinoma 1; PD-1, programmed cell death protein 1; PD-L1, programmed death-ligand 1; PRC2, Polycomb Repressive Complex 2; SS, synovial sarcoma; STS, soft tissue sarcoma; SU2C-SARC032, Stand Up To Cancer–Sarcoma Alliance for Research through Collaboration 032 clinical trial; SUZ12, suppressor of zeste 12 homolog; TCR-T, T-cell receptor-engineered T-cell therapy; TKI, tyrosine kinase inhibitor; TLS, tertiary lymphoid structure; TMB, tumor mutational burden; TME, tumor microenvironment; UPS, undifferentiated pleomorphic sarcoma; UV, ultraviolet.

Histotype	Expected Immunotherapy Sensitivity	Relevant Immune/Molecular Features	Current Evidence for Immunotherapy Use	Future Directions
UPS	Intermediate-to-higher sensitivity among common STSs, especially immune-inflamed tumors	Complex genomics; higher CD8^+^ infiltration in subsets; macrophage-rich TME; occasional TLSs; antigen presentation signatures	Responses observed in SARC028 and perioperative pembrolizumab/radiotherapy studies; strong translational rationale	Integrate TLSs, myeloid states, SDH/metabolic signatures, radiotherapy priming, and myeloid-targeting combinations
MFS	Potential sensitivity in inflamed subsets	Complex genomics; immune-infiltrated subsets; stromal remodeling and antigen presentation variability	Less trial-specific evidence than UPS/DDLPS, but biologically aligned with immune-inflamed complex genomic STSs	Prospective histotype-specific inclusion; spatial profiling; radiotherapy/ICI strategies
DDLPS	Variable; selected benefit with ICI combinations/neoadjuvant approaches	Complex copy-number alterations; immune heterogeneity; macrophage/stromal barriers; TLSs in specific subsets	Included in SU2C-SARC032; benefit appears context-dependent	Histotype-aware biomarkers; combine ICI with radiotherapy, anti-angiogenic or myeloid/stromal modulators
LMS	Generally low-to-variable sensitivity; uterine and non-uterine LMS may differ	Often immune-suppressive myeloid/stromal microenvironment; variable antigen presentation; rare MSI/MMR-deficient cases	ICI monotherapy usually modest; occasional exceptional responders	Separate uterine vs. extrauterine disease; explore MMR/TMB outliers and rational combinations
Angiosarcoma	Variable; higher sensitivity in selected UV-associated cutaneous tumors	UV-mutational signatures and higher TMB in some cutaneous scalp/face tumors; vascular biology	Responses to ICI reported in selected cases and cohorts	Separate UV-associated cutaneous from visceral/radiation-associated disease; evaluate TMB, immune infiltration, and vascular combinations
ASPS	Relatively high sensitivity to PD-1/PD-L1 blockade despite low TMB	ASPSCR1-TFE3 fusion; angiogenic biology; immune infiltration may be dynamically enhanced during therapy	Atezolizumab monotherapy has shown durable activity and represents the clearest approved ICI paradigm in STS	Define mechanisms of exceptional sensitivity; optimize sequencing with TKIs and local therapy; identify resistance markers
SS	Low sensitivity to ICI; high relevance for TCR-T in antigen-positive/HLA-eligible disease	SS18-SSX fusion; low TMB; frequent CTA expression, such as NY-ESO-1/MAGE-A4; immune-cold TME	Afami-cel approved for selected MAGE-A4+, HLA-A*02+ advanced synovial sarcomas; NY-ESO-1 TCR-T active in trials	Expand antigen repertoire; overcome HLA restriction; improve trafficking and persistence; combination with TME modulators
MRCL	Low ICI sensitivity; promising TCR-T activity in selected CTA-positive disease	FUS-DDIT3 fusion; low TMB; frequent CTA expression; immune-cold features	Clinical activity reported with NY-ESO-1 and MAGE-A4 TCR-T strategies in selected patients	Optimize conditioning, antigen thresholds, safety, and durability of ACT
MPNST	Generally limited, but biologically heterogeneous	PRC2/SUZ12/EED alterations in subsets; antigen presentation defects; macrophage-rich TME	ICI evidence limited and inconsistent	Epigenetic reversal of antigen presentation; myeloid/stromal targeting; define PRC2-loss immune states
Rhabdomyosarcoma and other rare STSs	Usually limited; selected immune-active cases possible	Often low TMB; pediatric/AYA biology; variable antigen presentation; spatial T-cell exclusion may occur	Evidence fragmented and mostly investigational	Subtype-specific trials; antigen-directed strategies; epigenetic and microenvironmental modulation

**Table 2:** Current landscape of predictive biomarkers for immunotherapy in soft tissue sarcomas (STSs), stratified according to their level of clinical validation, representative examples, current clinical utility, and principal limitations. Biomarkers are classified into three evidence tiers: (i) validated or clinically actionable biomarkers with established regulatory or clinical relevance in selected patient populations; (ii) promising translational biomarkers supported by reproducible associations with immunotherapy response or prognosis but lacking prospective clinical qualification; and (iii) exploratory biomarkers that provide mechanistic insights into tumor–immune interactions and may support future biological stratification and trial design but require external validation before clinical implementation. ASPS, alveolar soft part sarcoma; CAF, cancer-associated fibroblast; CD8+, cluster of differentiation 8-positive cytotoxic T lymphocyte; glyCAF, glycosylation-associated cancer-associated fibroblast state; MAGE-A4, melanoma-associated antigen A4; MMR, mismatch repair; MSI, microsatellite instability; ORR, objective response rate; PD-1, programmed cell death protein 1; PD-L1, programmed death-ligand 1; SAMSs, Sarcoma-Associated Microenvironmental States; SDH, succinate dehydrogenase; SE, sarcoma ecotype; SS, synovial sarcoma; STS, soft tissue sarcoma; TAM, tumor-associated macrophage; TCR, T-cell receptor; TMB, tumor mutational burden; TLS, tertiary lymphoid structure.

Evidence Tier	Examples	Current Utility	Key Limitations
Validated/clinically actionable	PD-L1 (ASPS treated with PD-1/PD-L1 blockade); MSI-high, MMR deficiency, very high TMB (pantumor; agnostic); MAGE-A4 expression with HLA-A*02 (SS treated with afami-cel)	Regulatory or tumor-agnostic relevance, or clinically meaningful evidence in defined subgroups	Rare in STS; not broadly predictive; requires appropriate testing and histotype context
Promising translational biomarkers	TLS, Sarcoma Immune Classes (B-cell rich), CD8^+^ T-cell infiltration, antigen presentation signatures, TCR clonality, PD-L1+ TAMs, composite immune-inflamed signatures	Repeated association with response or prognosis in translational studies, retrospective and exploratory prospective cohorts (e.g., SARC028)	Assessment not standardized; spatial heterogeneity; variable histotype distribution; consistent association with outcome but not yet prospectively validated as a selection biomarker
Exploratory/preclinical biomarkers	Sarcoma ecotypes (SE1-SE3), SAMS1/SAMS2 ecosystems, CAF/glyCAF states, endothelial/pericyte states, succinate/SDH-related metabolic signatures, spatial immune-exclusion metrics	Derived from single-cohort transcriptomic/single-cell analyses. Mechanistic and hypothesis-generating; useful for trial design and biological stratification	Need external validation, harmonized assays, prospective clinical testing, and threshold definition

**Table 3:** Main adoptive cellular therapy platforms under investigation or in clinical use for soft tissue sarcomas, their principal safety concerns, general management principles, and current clinical interpretation. T-cell receptor-engineered T-cell therapy represents the most clinically mature adoptive cellular therapy platform in sarcoma, particularly for antigen-positive and HLA-A*02-restricted synovial sarcoma and myxoid round cell liposarcoma. Chimeric antigen receptor T-cell- and natural killer cell-based platforms remain predominantly investigational, with ongoing efforts focused on improving antigen specificity, cellular persistence, tumor trafficking, and activity within the immunosuppressive tumor microenvironment. Abbreviations: ACT, adoptive cell therapy; CAR-NK, chimeric antigen receptor natural killer cell therapy; CAR-T, chimeric antigen receptor T-cell therapy; CRS, cytokine release syndrome; HLA, human leukocyte antigen; HLA-A*02, human leukocyte antigen A02 allele group; ICANS, immune effector cell-associated neurotoxicity syndrome; MRCL, myxoid round cell liposarcoma; NK, natural killer; STS, soft tissue sarcoma; TCR-T, T-cell receptor-engineered T-cell therapy; TME, tumor microenvironment.

Platform	Key Safety Concerns	Management Principles	Interpretive Note
TCR-T	CRS, cytopenias, febrile neutropenia, infection, lymphodepletion toxicity, transaminitis/inflammatory syndromes, rare neurotoxicity, off-target peptide recognition, antigen escape	Specialized monitoring; CRS grading; supportive care; tocilizumab/corticosteroids when indicated; infection prophylaxis; careful HLA and antigen selection	Most mature ACT platform in sarcoma; efficacy strongest in antigen-positive, HLA-A*02-restricted synovial sarcoma and MRCL
CAR-T	CRS, ICANS, cytopenias, infection, antigen heterogeneity, on-target/off-tumor toxicity, poor trafficking, exhaustion in hostile TME	Standard cellular therapy toxicity protocols; safety switches and dual targeting under investigation; careful antigen selection	Clinical feasibility demonstrated, but durable responses remain uncommon in STS
CAR-NK/next-generation cellular platforms	Potentially lower CRS risk but limited sarcoma-specific clinical experience; persistence and efficacy uncertain	Clinical-trial setting; monitor as immune effector cell therapy; optimize persistence and trafficking	Investigational; may help mitigate exhaustion and manufacturing constraints

## Data Availability

No new data were created. Data sharing is therefore not applicable.
